# Editorial: The known, the unknown, and the future of glutamate transporters

**DOI:** 10.3389/fncel.2022.1005834

**Published:** 2022-08-17

**Authors:** Zila Martinez-Lozada, Sandra J. Hewett, Francisco Zafra, Arturo Ortega

**Affiliations:** ^1^Department of Pediatrics, The Children's Hospital of Philadelphia, Philadelphia, PA, United States; ^2^Program in Neuroscience, Department of Biology, Syracuse University, Syracuse, NY, United States; ^3^Center of Molecular Biology Severo Ochoa, School of Science, Consejo Superior de Investigaciones Científicas, Universidad Autónoma de Madrid, Madrid, Spain; ^4^Instituto de Investigación Sanitaria del Hospital Universitario La Paz — IdiPAZ, Madrid, Spain; ^5^Department of Toxicology, Centro de Investigación y de Estudios Avanzados del Instituto Politécnico Nacional, Ciudad de México, Mexico

**Keywords:** glutamate, glutamate transport, excitatory amino acid transporter (EAAT), vesicular glutamate transport proteins, system x_c_^−^

Glutamate is the main excitatory neurotransmitter in the central nervous system (Fonnum, 1984). It is required for essentially all cognitive functions, however, is also a neurotoxin. Therefore, maintenance of the glutamate extracellular concentration involves tight control of its release and uptake. Several glutamate transport proteins contribute to this regulation. Vesicular glutamate transporters (VGLUTs) package glutamate into synaptic vesicles (Omote et al., 2011). The excitatory amino acid transporters (EAATs), under physiological conditions, remove glutamate from the synaptic cleft (Danbolt et al., 2016). Lastly, the cystine/glutamate exchanger, also known as system $x_{\text{c}}^{-}$, exports glutamate in exchange for cystine (Jabaudon et al., 1999; Warr et al., 1999; Featherstone and Shippy, 2008). In this Research Topic, we assemble a review of current literature and new research on these transporters.

## VGLUTs

Glutamate is packaged into synaptic vesicles *via* one of three VGLUTs. As part of our collection, Hori and Takamori describe a novel method to monitor glutamate transport *in living nerve terminals* using the rodent giant synapse, the calyx of Held. In addition, they discuss what is presently known about factors that alter the amount and rate of glutamate refilling of synaptic vesicles and the relevance of these findings to central nervous system disorders (Hori and Takamori).

## EAATs

Termination of glutamate signaling is mediated *via* uptake by one of five EAATs (Hediger et al., 2013). Not surprisingly, impaired expression and/or activity of EAATs have negative repercussions on health. EAAT2 (GLT-1a,b) is predominately expressed by astrocytes, although a small portion of GLT-1a can be found on axon terminals (Chen et al., 2004; Furness et al., 2008; Melone et al., 2009; Zhou et al., 2018).

In this article collection, Yeung et al. examined the expression of EAAT2 in several brain regions from *postmortem* tissue of patients with Alzheimer's disease (AD) or control (Yeung et al.). The authors found no significant change in EAAT2 density but did observe spatial differences in EAAT2 expression in AD tissue with less immunoreactivity detected in main astrocyte branches, especially on those surrounding neuronal cell bodies (Yeung et al.). Whether this altered expression pattern has implications for glutamate recycling in AD remains to be determined. Direct evidence for pathological loss of function of EAAT2 in a mouse model of Huntington's disease (HD) comes from the paper of Hirschberg et al. Therein they report that abnormal protein-protein interactions of mutant huntingtin (mHTT) with EAAT2 binding partners reduce glutamate uptake in striatal astrocytes and mediate some of the HD-associated deficits studied. Abnormalities in uptake and motor function were alleviated *via* over-expression of a C-terminal truncated EAAT2 protein (Hirschberg et al.).

Because most of the glutamate clearance (80–90%) is mediated by astrocytic EAAT2/GLT-1, the function of GLT-1 in other cell types has remained largely unexplored. In this collection, two groups report interesting findings concerning the physiological function of GLT-1 in neurons and oligodendrocytes using cell-specific knockout mouse lines. The Rosenberg group found that hippocampal slices prepared from neuronal GLT-1 KO (synGLT-1 KO) mice are more vulnerable to excitotoxicity than slices from wild-type mice (Rimmele et al.). Whether this is due to metabolic compromise, or a disturbance of glutamate homeostasis is still unknown. Meanwhile, the Fuss group reports that deletion of GLT-1 ($GLT1^{Plp}{}^{1}$*icKO* mice) in maturing oligodendrocytes leads to hypomyelination in the *corpus callosum* of male, but not female, mice (Thomason et al.). The exact mechanism responsible for these abnormalities is at present unknown.

Finally, the Robinson group used pharmacological inhibition of EAAT function to demonstrate their importance to arteriole patency (Jackson et al.), highlighting another important function of EAATs, which is to act as a bridge between local neuronal activity and increases in blood flow. One can speculate that the dysfunction of EAATs observed in several neuropathologies could negatively affect neurovascular coupling.

It is worth nothing that EAATs not only transport glutamate but act as anion channels (Wadiche et al., 1995; Bergles et al., 2002; Jen et al., 2005). Herein, Kovermann et al. review what is known about this function of the EAATs in both health and disease (Kovermann et al.). For example, they discuss the relationship between EAAT mutations that alter anion channel activity (but not glutamate uptake) resulting in chloride dyshomeostasis and symptoms of neurological disease.

## System ${\text{x}}_{\text{c}}^{-}$

System ${\text{x}}_{\text{c}}^{-}$ (Sx${}_{\text{c}}^{-}$) is a Na^+^-independent, Cl^−^ -dependent heteromeric amino acid transporter — formed by two polypeptides, xCT, the subunit responsible for the transport function, and 4F2hc, the subunit required for membrane localization — that functions physiologically to export glutamate while importing cystine in a 1:1 ratio (Bannai and Kitamura, 1980; Bannai, 1986). Astrocytes appear to be the main cell type expressing Sxc- in the mature brain (Zhang et al., 2014; Ottestad-Hansen et al., 2018). Sx${}_{\text{c}}^{-}$ activity contributes to the maintenance of redox homeostasis (Banjac et al., 2008), is important for the synthesis of glutathione (Sato et al., 1998), and is a major source of ambient extracellular glutamate *in vivo* (Baker et al., 2002; De Bundel et al., 2011) However, under pathological conditions, glutamate release through Sx${}_{\text{c}}^{-}$ contributes to neurological diseases/disorders [for review see Lewerenz et al. (2013)].

In this article collection, the Hermans group describes and validates a method using tritiated glutamate as a substrate for reversed transport to evaluate the activity of system x${}_{\text{c}}^{-}$ (Beckers et al.) both in cultured cells and in synaptosomal preparations. Meanwhile, Bentea et al. investigated the effect of genetic deletion of xCT on two models of Parkinson's disease (PD) (Bentea et al.). They found protection against proteasome inhibition-induced nigrostriatal degeneration, — but not MPTP-induced striatal toxicity — in xCT^−/−^ mice. Last but not least, He and Hewett evaluated the contribution of Sx${}_{\text{c}}^{-}$ to ischemic stroke (He and Hewett). Their results demonstrate that Sx${}_{\text{c}}^{-}$ contributes to cortical ischemic damage when blood flow is moderately but not severely reduced.

Altogether, in this Research Topic, the authors review current literature or provide original research centered on understanding more fully the physiological and/or pathophysiological function of glutamate transport. The work presented here highlights the need for further research on the myriad of roles glutamate transporters play in health and pathology. Therefore, we hope that this collection encourages additional research in this field.

## Author contributions

ZM-L produced the initial draft. SH, FZ, and AO further edited and provided additional content and references. All authors approved the final version for publication.

## Conflict of interest

The authors declare that the research was conducted in the absence of any commercial or financial relationships that could be construed as a potential conflict of interest.

## Publisher's note

All claims expressed in this article are solely those of the authors and do not necessarily represent those of their affiliated organizations, or those of the publisher, the editors and the reviewers. Any product that may be evaluated in this article, or claim that may be made by its manufacturer, is not guaranteed or endorsed by the publisher.
